# Social determinants and behaviors associated with overweight and obesity among youth and adults in a peri-urban area of Maputo City, Mozambique

**DOI:** 10.7189/jogh.11.04021

**Published:** 2021-03-27

**Authors:** Ivalda Macicame, António Prista, Klaus G Parhofer, Nílzio Cavele, Cremildo Manhiça, Sheila Nhachungue, Elmar Saathoff, Eva Rehfuess

**Affiliations:** 1 Instituto Nacional de Saúde, Maputo City, Mozambique; 2 Center for International Health (CIH), Ludwig-Maximilians-Universität München (LMU) Munich, Germany; 3 Universidade Pedagógica, Maputo City, Mozambique; 4 Medizinische Klinik IV – Grosshadern, Klinikum der Ludwig-Maximilians-Universität München (LMU), Munich, Germany; 5 Division of Infectious Diseases and Tropical Medicine, University Hospital, LMU, Munich, Germany; 6 German Centre for Infection Research (DZIF), partner site Munich, Munich, Germany; 7 Institute of Medical Information Processing, Biometry and Epidemiology, Pettenkofer School of Public Health, LMU Munich, Munich, Germany

## Abstract

**Background:**

Overweight and obesity are important risk factors for non-communicable diseases (NCDs) such as cardiovascular diseases (CVD), type 2 diabetes and certain cancers. NCDs are responsible for an increased number of deaths worldwide, including in developing countries. We aimed to determine the prevalence of overweight and obesity among youth and adults in a peri-urban area of Maputo city, Mozambique, and to assess their social and behavioral determinants.

**Methods:**

A cross-sectional study was conducted in a Health and Demographic Surveillance System (HDSS) area in Maputo city. We measured BMI and interviewed 15-64-year-old inhabitants to assess sociodemographic and behavioral characteristics using the STEPwise Approach methodology. A household wealth index was derived through Principal Component Analysis of various household assets and physical activity (PA) was measured using pedometers and accelerometers. Univariable and multivariable analyses were conducted to determine associations between overweight/obesity and social and behavioral determinants.

**Results:**

Among a total of 931 participants, the prevalence of overweight (BMI≥25 kg/m^2^) and obesity (BMI≥30 kg/m^2^) was 30.9% (95% confidence interval (CI) = 28.0, 33.9) and 12.6% (95% CI = 10.4, 14.7), respectively; one in every 10 youths and adults were underweight. Being female, older and living in a wealthier household were found to be significantly associated with overweight and obesity. Those with higher levels of education were found to have a reduced risk of being obese compared to those with no or lower levels of education. Behavioral risk factors (diet, alcohol and tobacco consumption and physical activity) did not significantly increase the risk of overweight and obesity.

**Conclusions:**

Overweight and obesity are highly prevalent in this peri-urban part of the Mozambican capital, where underweight is still present in youth and adults, confirming that the country is facing a double burden of malnutrition. Social determinants of health should be taken into consideration in the design and implementation of NCD prevention programs.

Globally, non-communicable diseases (NCDs), notably cardiovascular disease (CVD), diabetes, chronic respiratory disease and cancers, currently cause more deaths than all other causes combined [1]. The prevalence of overweight and obesity, which are important risk factors for NCDs such as CVD, type 2 diabetes and certain cancers, has more than doubled during the last three decades worldwide [1]. Although one of the objectives of the Global Action Plan for the prevention and control of NCDs is to tackle the modifiable risk factors for NCDs and their underlying social determinants through the creation of health-promoting environments, research on the social determinants of health is rarely done in low-income countries [2]. Monitoring the trends of overweight and obesity and understanding their determinants, particularly in urban settings from low-income countries, is crucial for achieving this objective [3].

In 2016 adults in low- and middle-income countries had almost twice the risk of dying from NCDs compared to those from high-income countries [4] and the prevalence of obesity in the African region was estimated to be about 15% in women and 6% in men [1].

According to the WHO, NCDs are currently responsible for approximately 32% of deaths in Mozambique [5]. Over the past few decades, the prevalence of overweight and obesity among Mozambican youth [6] and adults [7] has increased. According to the last STEPS survey, undertaken in 2014/2015, to assess risk factors for NCDs conducted in Mozambique, the prevalence of overweight increased from 18.3% to 30.5% in women and from 11.7% to 18.2% in men compared to the STEPS survey undertaken in 2005. Similarly, over the same time period the prevalence of obesity increased from 7% to 13% in women and from 2% to 5% in men. A higher prevalence was observed in urban than in rural areas [7,8].

Rapid population growth in urban settings, mostly through in-migration from rural areas to peri-urban areas, is leading people from low-income countries such as Mozambique to live in precarious conditions [6]. This rampant and unplanned urbanization, characterized by a lack of open space and an increase in criminality as distinctive features of African urban centers, is coinciding with decreasing physical activity levels [9] and increasing obesity and its comorbidities [10]. The objective of this study was to determine the prevalence of overweight and obesity among youth and adults in a peri-urban area of Maputo city, Mozambique, and to assess their social and behavioral determinants.

## METHODS

### Study population and sampling procedure

**Figure 1:**
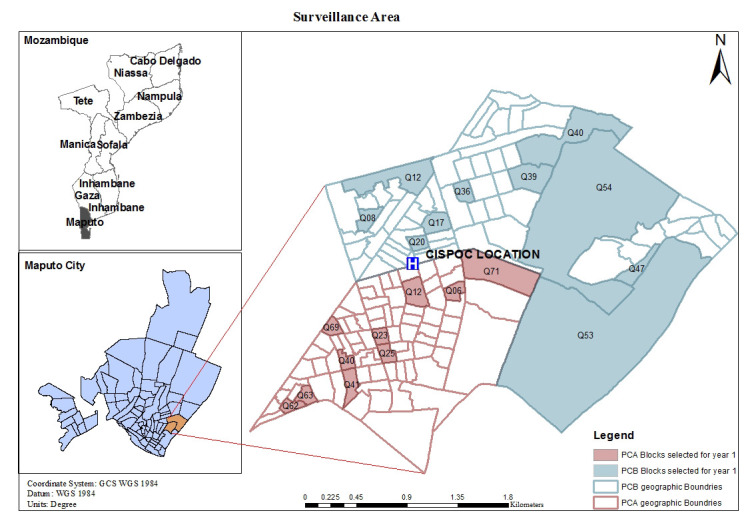
HDSS Polana Caniço surveillance area.

Mozambique is a low-income country located on the east coast of southern Africa. A Health and Demographic Surveillance System (HDSS) is being implemented in a peri-urban area of Maputo city, Polana Caniço, with about 92 000 inhabitants living in an area of 6.8 km^2^ [11]. The HDSS has enrolled approximately 15 000 inhabitants (from 20 randomly selected blocks) between January and June 2017. **Figure 1** shows the HDSS Polana Caniço surveillance area.

From October 2017 to April 2018 we recruited 15-64 year-old males and non-pregnant females from randomly selected households from the HDSS. Given the unknown prevalence of risk factors for NCDs specifically for Maputo city, a prevalence of 50% was used to calculate the required sample size in a population of 92000 inhabitants. Due to possible physiological / metabolic differences between men and women, the sample size was doubled in order to allow for gender-disaggregated analyses. Additionally, the sample was increased by 10% in order to compensate for the nature of the study (data collection at the community level) where blank responses and loss of quality of biological samples were likely to occur. This resulted in a sample size of 843 individuals, corresponding to a minimum of 282 households. To overcome issues related to household unavailability or refusals, an additional 282 households were randomly selected as replacements. Community leaders (secretariats of neighbourhoods, heads of blocks and other trusted individuals from the community) supported the study team with the community sensitization through six community meetings and door-to-door awareness-raising activities. Community leaders also scheduled the visit to the selected households. In each selected household, all individuals aged 15 to 64 years were eligible for enrolment into the study. After three attempted visits, if eligible individuals were not available, surrogate households were approached.

### Data collection

Two questionnaires – a household questionnaire and a demographic and risk factor questionnaire – were administered by trained interviewers in Portuguese or in a local language (XiChangana or Ronga); physical measurements were taken by the same trained interviewers.

The household questionnaire, based on Vyas and Kumaranayake [12], was completed by the head of household or his/her representative and included questions regarding household assets, type of hygiene facilities, construction materials and other relevant information to determine a setting-specific household wealth index. The demographic and risk factor questionnaire, based on the STEPwise Approach [13] and modified to fit the specific study objectives, was completed by each study participant. This covered questions related to age, gender, place of birth, education level, mother tongue, knowledge of Portuguese and occupation; as well as questions related to diet, physical activity and tobacco and alcohol consumption. Participant height was measured in centimeters using a stadiometer (SECA 2013, SECA Group, Hamburg, Germany) and weight in kilograms using a balance (OMRON HN289, OMRON HealthCare, Yangzhou, China). Participants with a Body Mass Index (BMI) of 30 kg/m^2^ or above were referred for nutritional counseling at a health facility, with all health facilities in the area offering this service. To measure physical activity, the number of steps and the time spent in moderate to vigorous physical activity (MVPA) were recorded using pedometers (STEPmvx, Guangdong, China) for seven consecutive days, except when sleeping and while taking a bath. No financial or material compensation was provided to participants.

This study was conducted according to the ethical principles described in the Helsinki Declaration and its revisions [14]. It was approved by the *Instituto Nacional de Saúde* (National Institute of Health - INS) Institutional Review Board and the National Health Bioethics Committee of Mozambique, as well as the Ethics Board of the Faculty of Medicine of Ludwig-Maximilians-Universität München, Germany. The voluntary participation of each adult household member was documented by written informed consent. For the voluntary participation of individuals between the ages of 15 and 17, a written assent form was administered to the minor and written informed consent was provided by the minor's legal representative. The findings of this study are being reported in accordance with the STROBE statement [15].

### Statistical analysis

**Figure 2:**
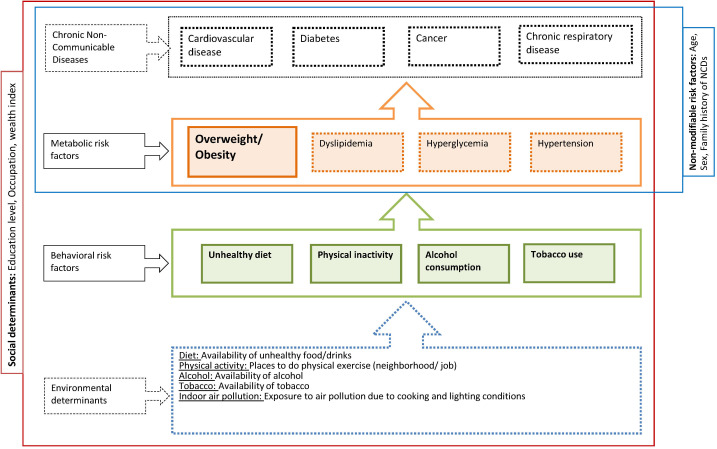
Conceptual framework of risk factors for overweight and obesity and related health outcomes.

A conceptual framework (**Figure 2**) was developed to describe the relationships between four NCDs (cardiovascular diseases, diabetes, cancer and chronic respiratory disease) and their underlying risk factors, including non-modifiable, metabolic and behavioral risk factors as well as their broader environmental and social determinants. With respect to behavioral risk factors, we focused on those for which the link with overweight and obesity can be considered causal, ie, an unhealthy diet, insufficient physical activity, alcohol consumption and tobacco use [16]. The analysis was mostly determined a priori, drawing on the conceptual framework and focusing on non-modifiable determinants, social determinants and behavioral risk factor as predictors of overweight and obesity.

Questionnaires were designed using the Open Data Kit (ODK) Collect 1.14.0 app (OpenRosa Consortium, University of Washington, Seattle, USA) and data were entered electronically using tablet computers (Samsung Galaxy TAB E 9.6 inches, Seoul, South Korea). Data were exported to an ODK aggregate 1.4.13 server using a mysql 5.7.1 database. Data checking and cleaning was conducted independently by a staff trained on quality assurance and control and by a statistician using Excel 2013 and R Studio version 3.4.2 (R Foundation, Boston, MA, USA). Data were subsequently exported to Stata version 15.1 (StataCorp LLC, College Station, Texas, USA) for analysis.

Socio-demographic characteristics of study participants are described using medians and inter quartile ranges (IQR) for continuous variables and proportions for categorical variables, for all participants and stratified by gender. A household wealth index was generated using a modification of an approach initially described by Filmer and Pritchett [17,18]. This method uses principal component analysis to combine data regarding ownership of various household assets, building materials of the house and other indicators of household wealth into a single index, which is then issued as a categorical variable (quintiles). Body Mass Index (BMI) calculated as weight (in kg) over (height (in m^2^), was categorized as underweight (<18.5 kg/m^2^), normal weight (18.5 to <25 kg/m^2^), overweight (≥25 to <30 kg/m^2^) and obese (≥30 kg/m^2^) according to the World Health Organization (WHO) [19]. The level of physical activity was determined by the number of steps and the time spent in moderate to vigorous physical activity (MVPA) per day using cutoff values of 10000 steps per day and 30 minutes per day, respectively. χ^2^-testing was used to examine differences in BMI distribution and behavioral risk factors for NCDs between males and females.

Associations of various potential risk factors with the binary outcome overweight (BMI≥25, which includes obese patients and is called “overweight+” below) were analyzed using log-link binomial regression and Poisson regression [20,21] with robust variance estimates to adjust for within household clustering. In a first step, univariable regressions adjusted for age and gender were used to identify factors whose association with overweight + had a *P*-value (*P*) ≤0.1 to be considered for inclusion into multivariable models. Based on the univariable results, three different multivariable models were developed according to the type of risk factor (socioeconomic factors, behavioral risk factors, behavioral and socioeconomic factors combined) shown in **Figure 2**. Decisions on which variables to retain in these multivariable models were guided by the Akaike Information Criterion (AIC) and the most parsimonious model was chosen. If two or more variables that qualified for inclusion were too collinear to be included in the same model (variance inflation factor ≥10) [22], the variable that resulted in the lowest AIC was used. A final model was constructed to combine the above identified factors into one model. Again the most parsimonious model according to the AIC was chosen. Gender, age and household wealth index were included in all multivariable models to adjust for potential confounding.

The procedures described above were also used to analyze the associations of various potential risk factors with the binary outcome obesity (BMI ≥30), using a more relaxed *P*-value of ≤0.2 for inclusion into multivariable models.

## RESULTS

**Figure 3:**
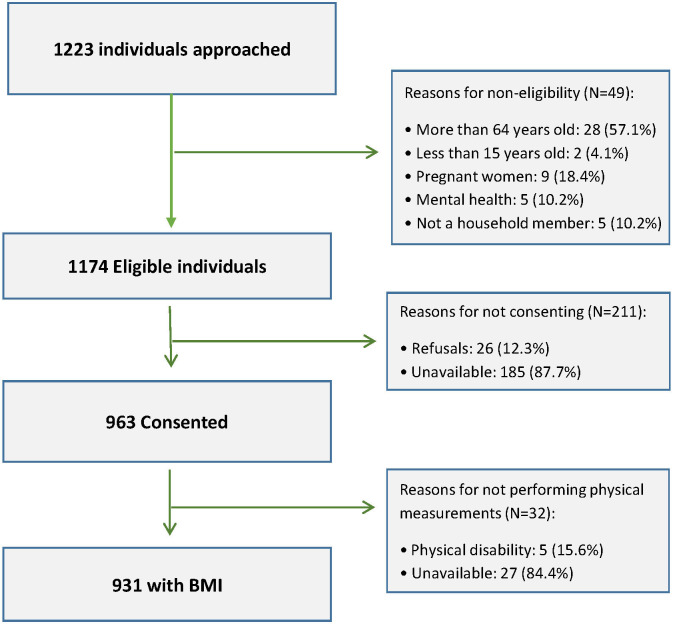
Screening and enrolment of participants.

Overall 1223 individuals living in 367 households (mean of three persons per household, with a minimum of one and a maximum of 13 adults per household) were approached, of whom 1174 individuals (96%) were eligible for inclusion in the study. Among the eligible participants, 963 (82%) – at least one member of the 367 households –consented to participate. From the consented participants 32 (3.3%) were excluded from this analysis for not having BMI data. Reasons for non-eligibility and for not consenting are presented in **Figure 3**. Socio-demographic characteristics of study participants are presented in **Table 1**. Among 931 participants with BMI data, 394 (42.3%) were males and 537 (57.7%) were females. The median age was 28 (IQR = 21, 41) years and half of the study participants (51%) were single. More than half of the participants (53.6% total, 61.9% for males and 47.5% for females) had at least completed primary education and 54.2% (69.9% for males and 42.3% for females) were employed. Portuguese, the official language in Mozambique, was considered as their mother tongue by 28.4% of the participants and 88% reported that they can speak, write and read Portuguese. A good command of Portuguese may play a role, as the dissemination of official health information in Mozambique tends to take place in Portuguese, the country’s official language.

**Table 1:** Socio-demographic characteristics of study participants (N = 931)

Characteristics	Total (N = 931)		Male (n = 394)		Female (n = 537)	
**N**	**%**	**n**	**%**	**n**	**%**
Age, years; median (IQR)	28 (21, 41)		27 (20, 38)		29 (21, 43)	
Age, years:	922		391		531	
15-24	372	(40.4)	165	(42.2)	207	(39.0)
25-34	223	(24.2)	104	(26.6)	119	(22.4)
35-44	133	(14.4)	57	(14.6)	76	(14.3)
45-54	121	(13.1)	39	(10.0)	82	(15.4)
55-64	73	(7.9)	26	(6.7)	47	(8.5)
Religion:	869		364		505	
Muslim	30	(3.5)	16	(4.2)	13	(2.5)
Zion	132	(15.2)	42	(11.1)	82	(15.6)
Christian Catholic	168	(19.3)	78	(20.7)	86	(16.3)
Christian Protestant	474	(54.6)	175	(46.4)	293	(55.6)
No religion	65	(7.5)	39	(10.3)	25	(4.7)
Marital status:	923		391		532	
Single	471	(51.0)	216	(55.2)	255	(47.9)
Marriage/Consensual union	393	(42.6)	165	(42.2)	228	(42.9)
Divorced/widowed	59	(6.4)	10	(2.6)	49	(9.2)
Mother tongue:	922		390		532	
Portuguese	262	(28.4)	116	(29.7)	146	(27.4)
Bitonga	25	(2.7)	11	(2.8)	14	(2.6)
Xichangana	458	(49.7)	169	(43.3)	289	(54.3)
Xisena	4	(0.4)	1	(0.3)	3	(0.6)
Xirhonga	20	(2.2)	7	(1.8)	13	(2.4)
Xichope	6	(0.7)	1	(0.3)	5	(0.9)
Xitshwa	35	(3.8)	19	(4.9)	16	(3.0)
Ndau	22	(2.4)	16	(4.1)	6	(1.1)
Other	90	(9.8)	50	(12.8)	40	(7.5)
Education:	922		391		531	
Primary or lower	428	(46.4)	149	(38.1)	279	(52.5)
Secondary or higher	494	(53.6)	242	(61.9)	252	(47.5)
Speak, write and read Portuguese:	864		380		484	
Yes	760	(88.0)	352	(92.6)	408	(84.3)
No	104	(12.0)	28	(7.4)	76	(15.7)
Occupation:	830		362		468	
Unemployed/ retired	183	22.1	54	14.9	129	27.6
Housewife	51	6.1	0	0	51	10.9
Full-time Student	146	17.6	56	15.5	90	19.2
Employed	450	54.2	252	69.9	198	42.3
Household wealth index score, quintiles:	819		346		473	
Lowest	109	13.3	55	15.9	54	11.4
Low	147	18.0	61	17.6	86	18.2
Medium	174	21.3	76	22.0.	98	20.7
High	192	23.4	78	22.5	114	24.1
Highest	197	24.1	76	22.0	121	25.6

IQR – interquartile range

Overall, the median BMI was 22.5 (IQR = 20.1, 26.2) kg/m^2^. BMI distribution and behavioral characteristics of study participants are presented in **Table 2** with 30.9% (95% CI = 28.0, 33.9) and 12.6% (95% CI = 10.4, 14.7) of the study participants being overweight + and obese, respectively. Females were characterized by a higher prevalence of overweight and obesity (42.5% overweight + and 19.4% obese) than males (*P* < 0.0001).

**Table 2:** Behavioral characteristics and BMI distribution of participants (N = 931)

Characteristics	Total (931)		Male (394)		Female (523)		*P*-value (χ^2^)
**N**	**%**	**n**	**%**	**n**	**%**	
BMI	931		394		537		<0.0001
Median (IQR):	22.5 (20.1, 26.2)		21.2 (19.3, 23.6)		23.7 (20.8, 28.3)		
<18.5	91	9.8	57	14.4	34	6.3	
18.5-24,9	552	59.3	277	70.3	275	51.2	
25-29.9	171	18.4	47	13.0	124	23.1	
≥30	117	12.6	13	3.3	104	19.4	
Consumption of fruit per week, days:	922		389		533		
Median (IQR)	2 (0, 3)		2 (0, 3)		2 (0, 3)		
Never	248	26.9	107	27.5	141	26.5	0.4284
1-3 d a week	488	52.9	197	50.6	291	54.6	
4-7 d a week	186	20.2	85	21.9	101	19.0	
Consumption of vegetables per week, days:	892		372		520		
Median (IQR)	4 (3, 6.5)		4 (3, 6)		4 (3, 7)		
Never	15	1.7	10	2.7	5	1.0	0.1107
1-3 days a week	385	43.2	164	44.1	221	42.5	
4-7 days a week	492	55.2	198	53.2	294	56.5	
Consumption of processed foods per week:	921		390		531		0.0867
Never	311	33.8	132	33.9	179	33.7	
Less than once per week	369	40.1	145	37.2	224	42.2	
1-4 times per week	175	19.0	76	19.5	99	18.6	
Daily/ Almost daily	66	7.2	37	9.5	29	5.5	
Consumption of soft drinks per week:	894		373		521		0.0080
Never	507	56.7	202	54.2	305	58.5	
Less than once per week	187	20.9	71	19.0	116	22.3	
1-4 times per week	145	16.2	66	17.7	79	15.2	
Daily/Almost daily	55	6.2	34	9.1	21	4.0	
Tobacco consumption	923		390		533		<0.0001
Never consumes	835	90.5	319	81.8	516	96.8	
Consumes weekly	36	3.9	28	7.2	8	1.5	
Consumes daily	52	5.6	43	11.0	9	1.7	
Alcohol consumption:	922		390		532		<0.0001
Never consumed alcohol	397	43.1	113	29.0	284	53.4	
Did not consume alcohol last year	95	10.3	34	8.7	61	11.5	
Consumes less than once per week	380	41.2	205	52.6	175	32.9	
Consumes daily to at least once per week	50	5.4	38	9.7	12	2.3	
Number of steps per day	537		228		309		
Median (IQR)	8136 (5409, 11 638)		10 166 (6668, 13 866)		6819 (4634, 9897)		
<10 000	347	64.6	110	48.3	237	76.7	<0.0001
≥10 000	190	35.4	118	51.7	72	23.3	
Time spent in MVPA per day, minutes	537		228		309		
Median (IQR)	18,0 (6.2, 35.0)		24.8 (10.2, 46.5)		14.0 (3.8, 28.7)		
<60	360	67.0	128	56.1	232	75.1	<0.0001
≥60	177	33.0	100	43.9	77	24.9	

BMI – body mass index, IQR – interquartile range, MVPA – moderate to vigorous physical activity

The median consumption of fruit and vegetables was on 2 days and 4 days per week, respectively; 26.9% and 1.7% of participants never eat fruit and vegetables in a week, respectively. Daily or almost daily consumption of processed foods and soft drinks were reported by 7.2% and 6.2% of participants, respectively. There were no statistically significant differences regarding the consumption of fruit and vegetables, processed foods and soft drinks between females and males. Daily tobacco consumption was reported by 5.6%, and 5.4% of participants reported consuming alcohol daily or at least once per week, with males consuming more tobacco and alcohol than females (*P* < 0.0001). Of those who correctly used pedometers to measure physical activity (ie, throughout the day and over seven days), 64.8% walked less than the recommended 10000 steps per day, with a median (IQR) of 8136 (5409, 11638) steps per day, while 67.4% spent less than 60 minutes of MVPA per day, with a median (IQR) of 18 (6.2, 35) minutes of MVPA per day. Females engaged less in physical activity than males (*P* < 0.0001).

Table 3 presents the findings of the univariable modelling. After adjusting for age, females were 2.57 times (95% CI = 2.02 to 3.27) more likely to be overweight + than males and after adjusting for gender the risk of being overweight + increased 1.03 times (95% CI = 1.03, 1.04) with each year of age. The highest household wealth index was found to be associated with a 1.92 fold (95% CI = 1.32, 2.79) increased risk of being overweight + compared to the lowest household wealth index category (*P* = 0.0006). Married and employed people were 1.45 (95% CI = 1.13, 1.87) and 1.51 (95% = 1.19, 1.92) times more likely to be overweight + than singles and unemployed participants, respectively. After adjusting for age and gender, the level of education, having Portuguese as one’s mother tongue and being able to speak, write and read Portuguese did not show an association with overweight+.

**Table 3:** Associations of overweight+ (BMI≥25) with socioeconomic and behavioral characteristics, univariable models and models adjusted for gender and age

				Univariable*				Adj. for gender & age		
**Covariate**	**N**	**Evts.**	**% Evts.**	**RR**		**95% CI**	***P*-value**	**RR**	**95% CI**	***P*-value**
Gender (N = 922):										
Male	391	59	15.09	1.00		–	–	1.00	–	–
Female	531	223	42.00	2.78		(2.18, 3.56)	<0.0001	2.57	(2.02, 3.27)	<0.0001
Age:										
Per year	–	–	–	1.04		(1.03, 1.04)	<0.0001	1.03	(1.03, 1.04)	<0.0001
Household wealth index score, quintiles (N = 818):										
Very low	109	26	23.85	1.00		–	–	1.00	–	–
Low	146	40	27.40	1.15		(0.72, 1.83)	0.5622	1.20	(0.78, 1.86)	0.4127
Medium	174	48	27.59	1.16		(0.75, 1.78)	0.5093	1.23	(0.83, 1.83)	0.2997
Medium/high	192	58	30.21	1.27		(0.82, 1.96)	0.2906	1.35	(0.90, 2.01)	0.1421
Highest	197	83	42.13	1.77		(1.18, 2.65)	0.0062	1.92	(1.32, 2.79)	0.0006
Education (N = 921):										
Primary or lower	427	171	40.05	1.00		–	–	1.00	–	–
Secondary or higher	494	110	22.27	0.56		(0.45, 0.68)	<0.0001	1.03	(0.81, 1.30)	0.8250
Marital status (N = 922):										
Single	471	92	19.53	1.00		–	–	1.00	–	–
Married	392	159	40.56	2.08		(1.63, 2.65)	<0.0001	1.45	(1.13, 1.87)	0.0035
Divorced/widow(ed)	59	31	52.54	2.69		(1.96, 3.70)	<0.0001	1.18	(0.84, 1.66)	0.3517
Occupation (N = 922):										
Unemployed/retired	183	50	27.32	1.00		–	–	1.00	–	–
Housewife	51	19	37.25	1.36		(0.87, 2.14)	0.1758	1.10	(0.73, 1.68)	0.6445
Student	146	21	14.38	0.53		(0.32, 0.85)	0.0095	0.94	(0.57, 1.54)	0.8058
Employed	449	159	35.41	1.30		(0.99, 1.70)	0.0623	1.51	(1.19, 1.92)	0.0008
Missing data	93	33	35.48	1.30		(0.89, 1.89)	0.1715	1.48	(1.05, 2.07)	0.0236
Can speak, write and read Portuguese (N = 922):										
No	103	40	38.83	1.00		–	–	1.00	–	–
Yes	760	218	28.68	0.74		(0.56, 0.97)	0.0299	1.20	(0.93, 1.56)	0.1627
Missing data	59	24	40.68	1.05		(0.72, 1.53)	0.8109	0.76	(0.53, 1.09)	0.1344
Portuguese as the mother language (N = 922):										
No	660	232	35.15	1.00		–	–	1.00	–	–
Yes	262	50	19.08	0.54		(0.42, 0.71)	<0.0001	0.89	(0.67, 1.19)	0.4469
Consumption of fruit per week (N = 919):										
Per number of days of the week	–	–	–	1.04		(1.00, 1.09)	0.0647	1.07	(1.03, 1.11)	0.0004
Consumption of fruit per week (N = 919):										
Never	247	72	29.15	1.00		–	–	1.00	–	–
1-3 days a week	486	146	30.04	1.03		(0.81, 1.31)	0.8041	1.18	(0.95, 1.47)	0.1365
4-7 days a week	186	64	34.41	1.18		(0.89, 1.56)	0.2484	1.44	(1.11, 1.86)	0.0052
Consumption of vegetables per week (N = 891):										
Per number of days of the week	–	–	–	1.02		(0.98, 1.07)	0.3134	1.00	(0.95, 1.04)	0.8330
Consumption of vegetables per week (N = 891)										
Never	15	2	13.33	1.00		–	–	1.00	–	–
1-3 days a week	385	118	30.65	2.30		(0.63, 8.33)	0.2050	1.91	(0.54, 6.77)	0.3142
4-7 days a week	491	160	32.59	2.44		(0.67, 8.92)	0.1761	1.84	(0.51, 6.57)	0.3495
Consumption of processed food per week (N = 919):										
Never	310	96	30.97	1.00	–			1.00		
Less than once per week	368	126	34.24	1.11	(0.89, 1.38)		0.3708	1.13	(0.91, 1.39)	0.2686
1-4 times per week	175	49	28.00	0.90	(0.68, 1.21)		0.4969	1.15	(0.88, 1.51)	0.3055
Daily/almost daily	66	10	15.15	0.49	(0.26, 0.92)		0.0266	0.80	(0.44, 1.45)	0.4567
Consumption of soft drinks per week (N = 893):										
Never	507	174	34.32	1.00	–		–	1.00	–	–
Less than once per week	186	58	31.18	0.91	(0.72, 1.15)		0.4237	1.02	(0.82, 1.25)	0.8863
1-4 times per week	145	33	22.76	0.66	(0.48, 0.92)		0.0152	0.86	(0.64, 1.16)	0.3345
Daily/almost daily	55	14	25.45	0.74	(0.47, 1.17)		0.2005	1.08	(0.69, 1.70)	0.7249
Tobacco consumption (N = 920):										
Never consumed	833	270	32.41	1.00	–		–	1.00	–	–
Consumes weekly	36	8	22.22	0.69	(0.38, 1.24)		0.2113	0.95	(0.55, 1.63)	0.8462
Consumes daily	51	4	7.84	0.24	(0.10, 0.57)		0.0013	0.27	(0.11, 0.68)	0.0049
Alcohol consumption (N = 919):										
Never consumed alcohol	396	121	30.56	1.00	–		–	1.00	–	–
Did not consume alcohol last year	94	37	39.36	1.29	(0.97, 1.72)		0.0841	1.16	(0.91, 1.48)	0.2304
Consumes less than once per week	379	116	30.61	1.00	(0.80, 1.25)		0.9883	1.23	(1.00, 1.50)	0.0485
Consumes daily to at least once per week	50	8	16.00	0.52	(0.25, 1.09)		0.0825	0.90	(0.45, 1.79)	0.7663
Mean of steps per day (N = 895):										
≤10 000	347	117	33.72	1.00	–		–	1.00	–	–
>10 000	189	52	27.51	0.82	(0.62, 1.08)		0.1562	0.96	(0.75, 1.24)	0.7779
Missing data	359	111	30.92	0.92	(0.74, 1.14)		0.4350	0.95	(0.78, 1.15)	0.5783
Mean of MVPA per day (N = 895):										
60 min or less	359	117	32.59	1.00	–		–	1.00	–	–
Above 60 min	177	52	29.38	0.90	(0.70, 1.17)		0.4296	1.16	(0.91, 1.49)	0.2245
Missing data	359	111	30.92	0.95	(0.77, 1.18)		0.6298	1.00	(0.82, 1.22)	0.9966

N – number of patients/observations, Evts. – number of overweight and obese patients (BMI>-25), % Evts.- percentage of overweight and obese patients, RR – risk ratio, CI – confidence interval

*Associations analyzed using log-link binomial regression with robust variance estimates to adjust for within household clustering.

Regarding behavioral risk factors, the consumption of fruit was associated with a 1.07 (95% CI = 1.03, 1.11) times increased risk of overweight + for each additional day (*P* = 0.0004), in contrast to the consumption of processed foods and soft drinks that showed no association with overweight+. Tobacco consumption was found to reduce the risk of being overweight+ (*P* = 0.0049), while alcohol consumption and physical activity were not significantly associated with overweight+.

Appendix S1 in the **Online Supplementary Document** presents the associations of obesity with socioeconomic and behavioral characteristics. Associations were similar to those described for overweight+, with statistically significant associations between obesity and age, gender and household wealth index and no significant associations for diet, tobacco and physical activity.

**Table 4** presents the results for the final multivariable models for overweight+. These show that females were 2.38 times (95% CI = 1.84, 3.06) more likely to be overweight + than males and that the risk of becoming overweight + increased 1.03 times (95% CI = 1., 1.03) with every additional year of age. With respect to household wealth, only the highest wealth index category was significantly associated with overweight+, with a 1.98 times (95% CI = 1.38, 2.83) increased risk of overweight + compared to the lowest household wealth index category. Being married and being employed were associated with 1.45 times (95% CI = 1.13,1.86) and 1.33 times (95% CI = 1.04 to 1.69) the risk of overweight+, respectively. Consumption of fruit was associated with a1.05 times (95% CI = 1.01, 1.09) increased risk of overweight + while daily tobacco consumption was associated with a reduced risk of being overweight+.

**Table 4:** Multivariable association between overweight + and socioeconomic and behavioral characteristics in Maputo city, Mozambique (N = 817)

				Univariable			Multivariable		
**Covariate**	**N**	**Evts.**	**% Evts.**	**RR**	**95% CI**	***P*-value**	**RR**	**95% CI**	***P*-value**
Gender:									
Male*	345	55	15.94	1.00	–	–	1.00	–	–
Female	472	200	42.37	2.66	(2.06, 3.43)	0.0000	2.38	(1.84, 3.06)	<0.0001
Age:									
Per year	–	–	–	1.03	(1.03, 1.04)	0.0000	1.03	(1.02, 1.03)	<0.0001
Household wealth index, quintiles:									
Very low*	109	26	23.85	1.00	–	–	1.00	–	–
Low	146	40	27.40	1.15	(0.72, 1.83)	0.5622	1.29	(0.86, 1.95)	0.2161
Medium	174	48	27.59	1.16	(0.75, 1.78)	0.5093	1.30	(0.89, 1.89)	0.1758
Medium/high	192	58	30.21	1.27	(0.82, 1.96)	0.2906	1.35	(0.92, 1.98)	0.1206
Highest	196	83	42.35	1.78	(1.18, 2.67)	0.0058	1.98	(1.38, 2.83)	0.0002
Marital status:									
Single*	402	81	20.15	1.00	–	–	1.00	–	–
Married	357	143	40.06	1.99	(1.54, 2.56)	0.0000	1.45	(1.13, 1.86)	0.0033
Divorced/widow(ed)	58	31	53.45	2.65	(1.92, 3.66)	0.0000	1.21	(0.87, 1.68)	0.2557
Occupation:									
Retired/unemployed*	160	47	29.37	1.00	–	–	1.00	–	–
Housewife	45	16	35.56	1.21	(0.74, 1.97)	0.4412	0.96	(0.61, 1.52)	0.8737
Student	132	19	14.39	0.49	(0.30, 0.81)	0.0054	0.87	(0.52, 1.45)	0.5956
Employed	402	142	35.32	1.20	(0.91, 1.60)	0.2020	1.33	(1.04, 1.69)	0.0217
Missing data	78	31	39.74	1.35	(0.93, 1.96)	0.1108	1.54	(1.12, 2.12)	0.0084
Consumption of fruit per week:									
Per number of days of the week	–	–	–	1.04	(1.00, 1.09)	0.0602	1.05	(1.01, 1.09)	0.0175
Tobacco consumption:									
Never consumed*	743	244	32.84	1.00	–	–	1.00	–	–
Consumes weekly	31	8	25.81	0.79	(0.44, 1.40)	0.4127	0.89	(0.52, 1.51)	0.6584
Consumes daily	43	3	6.98	0.21	(0.08, 0.59)	0.0032	0.25	(0.09, 0.72)	0.0102

N – number of observations, Evts. – number of events, % Evts. – (N-events/N) × 100, RR – risk ratio, CI – confidence interval

*Reference stratum.

Appendix S2 in the **Online Supplementary Document** presents the results for the final multivariable models for obesity where, similar to overweight, being female, increasing age and a greater household wealth index were associated with a higher risk of obesity. Higher levels of education were associated with a significantly reduced risk of being obese.

## DISCUSSION

### Key findings

Previous studies carried out in Mozambique have described an increase of overweight and obesity prevalence, especially in urban areas [6-8,10]. The purpose of this study was to determine the prevalence of overweight and obesity and their association with social and behavioral determinants in the capital city of Mozambique, where urbanization is progressing rapidly and disorderly, with a visible reduction of recreation spaces and no active promotion of healthy lifestyle habits. We found that among youth and adults from the peri-urban area of Maputo city, one in every three is overweight and more than one in every ten is obese. Females, the elderly and people with greater household wealth have a significantly increased risk of being overweight and obese compared to males, younger people and individuals with lower household wealth index. As documented in previous studies [23-25], tobacco consumption is associated with a reduced risk of being overweight, and those with higher levels of education have a reduced risk of obesity compared to those with lower levels of education. Put simply, this suggests that – in this specific context of a peri-urban area of Maputo – being wealthier increases the risk of certain unhealthy lifestyle habits that enhance the risk of gaining weight, whereas being more educated appears to counteract the risk of gaining weight.

### Locating findings in the literature

The high levels of overweight and obesity found in this study are in accordance with data from Mozambique [6-8] and other SSA countries [26-31], and show once again that even countries with low levels of national wealth and urbanization such as Mozambique are facing the burden of overweight and obesity [32].

Similar to findings from the Mozambican STEPS surveys [7,8] and from other African countries [27,28,31,33-35], being female and being older are strong predictors of overweight and obesity, with risks of overweight and obesity being 2 times and more than 5 times greater among women compared to men, respectively. This difference between sexes has been previously explained by gender differences in metabolism and hormonal balance [36], among other cultural and behavioral reasons.

In agreement with previous studies that showed an association between household wealth and overweight in Africa, including in Mozambique [7,8,27,28,32,33,37,38], our study shows a strong association of household wealth with overweight and obesity, where participants in households with the highest household wealth index have an almost two times and a five times elevated risk of being overweight or obese respectively, compared to those from households with the lowest household wealth index.

Our results showed that married individuals are more likely to be overweight than singles - a finding that does not differ from previous studies from SSA [30,39].

In line with a previous study conducted in other SSA countries [30], our findings showed that higher levels of education seem to be protective against obesity, which might be related to the fact that more educated people are more informed regarding NCDs and associated risk factors, as well as health-protective behaviors. In contrast, those that are employed are more likely to be overweight compared to unemployed youth and adults, which might be explained by the fact that employed people from this study were more likely to consume processed and often high-energy foods.

Our findings did not show a decreased risk of overweight and obesity in those with a higher consumption of fruit and vegetables [40] as we would have expected. Conversely, it showed an increased risk of being overweight with an increase in the number of days on which fruit and vegetables are consumed.

Even in less urbanized countries such as Mozambique, urban residents purchase most of their food, whereas rural residents base their diet primarily on family farming. City dwellers from Maputo buy 92% of their food, which increases the probability of consuming pre-processed high energy foods [41]. Soft drinks and fast food are usually related to obesity due to their high energy content [42], however, this study did not find any association between BMI and consumption of processed food and consumption of soft drinks. Thus, the study documents the frequency of consumption of “junk food”, which can be used as a baseline for monitoring the dynamics of change in diet and can also provide evidence to the development of policies for the reduction of saturated fatty acids and trans-fats in Mozambique [5].

Physical activity is commonly described as a protective factor for overweight and obesity [43], including in SSA [27]. Although the study population showed low levels of physical activity (particularly among women), we did not find any association between physical activity and BMI. The relationship between BMI and physical activity is influenced by a complex combination of factors such as time spent watching television, environmental conditions to practice physical activity, urban crime and other predictors of physical inactivity [44], which are likely to play a role in explaining this result.

Studies from other African countries addressed the perception of ideal body image, where overweight individuals were found to be perceived as healthier, wealthier or prettier [35,39,45,46], making the problem of overweight and obesity even more complex in such environments. Understanding the social and behavioral function and predictors of overweight and obesity in the local context is crucial for designing and implementing interventions to reduce the prevalence of overweight and obesity in Mozambique and in other developing countries and, consequently, to prevent NCDs. Meanwhile, studies regarding cultural factors, beliefs and perceptions about body weight are needed in order to develop culturally appropriate new policies and to adapt existing ones to the local context in order to achieve the goals stated in the Global Action Plan for NCDs and the Sustainable Development Goals.

### Strengths and limitations

One of the major limitations of this study is its cross-sectional design that does not allow us to infer causality. Although the exclusion of 32 participants (3.3% of those who were eligible and provided informed consent) without anthropometric measurements could have been an important shortcoming, it is unlikely to have had a big influence on our effect estimates. As previous data described multiparous women to be at a higher risk of increased BMI [47], the fact that our study was unable to collect information regarding parity represents another limitation. We also recognize limitations related to the diet component of our study: first, the consumption of fruit and vegetables was not measured per unit, but per number of days consuming fruit and vegetables, due to the difficulty of the interviewees to recall this in detail; second, as this was not a dietary survey and hence lacks information regarding the consumption of saturated fat, animal protein and sugar, important elements of the diet of the population under study [48].

Nevertheless, this study reports substantial and reliable data regarding BMI from participants in a peri-urban developing country setting. With rapid urbanization Such peri-urban populations will represent the majority of the population in countries like Mozambique in the near future. Although this paper was focused on overweight and obesity, it also shows that underweight should not be neglected in adults (1 in every 10 adults were underweight), especially due to the high burden of HIV and tuberculosis [49]. Other strengths of this study are related to its methodology: a large sample size was achieved, allowing comparison of groups; context-sensitive wealth index quintiles were obtained through robust analysis; validated data collection tools from the STEPS survey were adapted to the local context; and objective measurements (using pedometers and accelerometers) were used to describe physical activity.

## CONCLUSION

In summary, the high prevalence of overweight and obesity and the low levels of physical activity and low intake of fruits and vegetables in peri-urban areas of Maputo city are of considerable public health concern. The statistically significant relationship between overweight/obesity and female sex, older age and higher household wealth highlights the need for focused and culturally appropriate interventions in this population to reduce the burden of overweight and obesity in Maputo city, Mozambique. NCD prevention and control measures focused on individual lifestyle and environmental factors to promote a healthier diet and increased physical activity are urgently needed.

## Additional material

Online Supplementary Document
