# Supplementary material for: Social determinants and behaviors associated with overweight and obesity among youth and adults in a peri-urban area of Maputo City, Mozambique
Source: J Glob Health. 2021 Mar 27;11:04021. doi: 10.7189/jogh.11.04021 (PMC8038757; doi:10.7189/jogh.11.04021)
Supplement: Online Supplementary Document [file jogh-11-04021-s001.pdf]

Table S1. Associations of obesity (BMI>=30) with socioeconomic and behavioral characteristics, univariable models and models adjusted for gender and age

				Univariable			Adj. for gender & age		
**Covariate**	**N**	**Evts.**	**% Evts.**	**RR**	**95% CI**	**p-value**	**RR**	**95% CI**	**p-value**
**Gender (N = 922)**									
**Male**	391	12	3.07	1.00	- -	-	1.00	- -	-
**Female**	531	102	19.21	6.26	(3.53 to 11.09)	<0.0001	5.59	(3.17 to 9.86)	<0.0001
**Age (per year)**	-	-	-	1.05	(1.04 to 1.06)	<0.0001	1.05	(1.03 to 1.06)	<0.0001
**Household wealth index, quintiles (N = 818)**									
**Very low**	109	5	4.59	1.00	- -	-	1.00	- -	-
**Low**	146	21	14.38	3.14	(1.22 to 8.06)	0.0176	3.34	(1.35 to 8.27)	0.0093
**Medium**	174	24	13.79	3.01	(1.22 to 7.42)	0.0169	3.24	(1.35 to 7.77)	0.0085
**Medium/high**	192	17	8.85	1.93	(0.73 to 5.08)	0.1829	2.11	(0.84 to 5.31)	0.1136
**Highest**	197	35	17.77	3.87	(1.59 to 9.42)	0.0028	4.34	(1.84 to 10.21)	0.0008
**Education (N = 921)**									
**Primary or lower**	427	82	19.20	1.00	- -	-	1.00	- -	-
**Secondary or higher**	494	32	6.48	0.34	(0.23 to 0.50)	<0.0001	0.75	(0.47 to 1.19)	0.2256
**Marital status (N = 922)**									
**Single**	471	35	7.43	1.00	- -	-	1.00	- -	-
**Married**	392	66	16.84	2.27	(1.57 to 3.27)	<0.0001	1.34	(0.91 to 1.97)	0.1377
**Divorced/ widow(ed)**	59	13	22.03	2.97	(1.75 to 5.03)	<0.0001	0.88	(0.48 to 1.62)	0.6768
**Occupation (N = 922)**									
**Unemployed/retired**	183	25	13.66	1.00	- -	-	1.00	- -	-
**Housewife**	51	6	11.76	0.86	(0.37 to 2.00)	0.7282	0.66	(0.29 to 1.50)	0.3159
**Student**	146	4	2.74	0.20	(0.07 to 0.56)	0.0021	0.44	(0.16 to 1.23)	0.1196
**Employed**	449	68	15.14	1.11	(0.74 to 1.67)	0.6219	1.41	(0.96 to 2.06)	0.0771
**Missing data**	93	11	11.83	0.87	(0.44 to 1.69)	0.6728	1.00	(0.54 to 1.88)	0.9879
**Can speak, write and read Portuguese (N = 922)**									
**No**	103	16	15.53	1.00	- -	-	1.00	- -	-
**Yes**	760	86	11.32	0.73	(0.44 to 1.19)	0.2090	1.46	(0.90 to 2.36)	0.1230

**Missing data**	59	12	20.34	1.31	(0.68 to 2.53)	0.4221	0.85	(0.45 to 1.59)	0.6069
**Portuguese as the mother language (N = 922)**									
**No**	660	99	15.00	1.00	- -	-	1.00	- -	-
**Yes**	262	15	5.73	0.38	(0.23 to 0.62)	0.0001	0.78	(0.45 to 1.36)	0.3802
**Consumption of Fruit per week (N = 919)**									
**(per number of days of the week)**	-	-	-	1.01	(0.93 to 1.10)	0.7643	1.05	(0.98 to 1.13)	0.1720
**Consumption of Fruit per week (N = 919)**									
**Never**	247	34	13.77	1.00	- -	-	1.00	- -	-
**1-3 days a week**	486	54	11.11	0.81	(0.55 to 1.18)	0.2656	0.97	(0.66 to 1.41)	0.8635
**4-7 days a week**	186	26	13.98	1.02	(0.64 to 1.61)	0.9481	1.35	(0.86 to 2.13)	0.1938
**Consumption of Vegetables per week (N = 891)**									
**(per number of days of the week)**	-	-	-	1.03	(0.96 to 1.12)	0.4059	0.99	(0.91 to 1.08)	0.8507
**Consumption of Vegetables per week (N = 891)**									
**Never**	15	1	6.67	1.00	- -	-	1.00	- -	-
**1-3 days a week**	385	47	12.21	1.83	(0.28 to 12.04)	0.5289	1.39	(0.23 to 8.45)	0.7190
**4-7 days a week**	491	65	13.24	1.99	(0.29 to 13.48)	0.4827	1.31	(0.21 to 8.15)	0.7735
**Consumption of Processed food per week (N = 919)**									
**Never**	310	43	13.87	1.00	- -	-	1.00	- -	-
**Less than once per week**	368	51	13.86	1.00	(0.68 to 1.47)	0.9964	1.01	(0.69 to 1.48)	0.9599
**1-4 times per week**	175	18	10.29	0.74	(0.45 to 1.23)	0.2479	1.02	(0.64 to 1.63)	0.9301
**Daily/almost daily**	66	2	3.03	0.22	(0.05 to 0.89)	0.0341	0.45	(0.11 to 1.78)	0.2547
**Consumption of Soft drinks per week (N = 893)**									
**Never**	507	70	13.81	1.00	- -	-	1.00	- -	-
**Less than once per week**	186	23	12.37	0.90	(0.57 to 1.41)	0.6314	1.05	(0.68 to 1.62)	0.8157
**1-4 times per week**	145	16	11.03	0.80	(0.47 to 1.35)	0.4031	1.17	(0.72 to 1.90)	0.5361
**Daily/almost daily**	55	3	5.45	0.40	(0.13 to 1.19)	0.0991	0.71	(0.24 to 2.07)	0.5267
**Tobacco consumption (N = 920)**									
**Never consumed**	833	110	13.21	1.00	- -	-	1.00	- -	-
**Consumes weekly**	36	1	2.78	0.21	(0.03 to 1.49)	0.1183	0.41	(0.07 to 2.40)	0.3221
**Consumes daily**	51	3	5.88	0.45	(0.16 to 1.22)	0.1157	0.65	(0.22 to 1.93)	0.4343
**Alcohol consumption (N = 919)**									
**Never consumed alcohol**	396	48	12.12	1.00	- -	-	1.00	- -	-

	N	Evts.	% Evts.	RR	95% CI	p-value	RR	95% CI	p-value
**Did not consume alcohol last year**	94	21	22.34	1.84	(1.16 to 2.92)	0.0093	1.64	(1.07 to 2.50)	0.0228
**Consumes less than once per week**	379	43	11.35	0.94	(0.63 to 1.38)	0.7404	1.28	(0.89 to 1.86)	0.1849
**Consumes daily to at least once per week**	50	2	4.00	0.33	(0.08 to 1.29)	0.1114	0.85	(0.23 to 3.22)	0.8156
**Mean of steps per day (N = 536)**									
**(per 10000 units)**	-	-	-	1.00	(1.00 to 1.00)	0.0835	1.00	(1.00 to 1.00)	0.4598
**Mean of MVPA per day (N = 536)**									
**(per 10 units)**	-	-	-	0.99	(0.98 to 1.00)	0.1949	1.00	(0.99 to 1.01)	0.8432
**Mean of steps per day (N = 895)**									
**10000 or less**	347	51	14.70	1.00	- -	-	1.00	- -	-
**Above 10000**	189	23	12.17	0.83	(0.52 to 1.31)	0.4227	1.02	(0.65 to 1.61)	0.9160
**Missing data**	359	39	10.86	0.74	(0.50 to 1.09)	0.1305	0.77	(0.53 to 1.12)	0.1678
**Mean of MVPA per day (N = 895)**									
**60 minutes or less**	359	52	14.48	1.00	- -	-	1.00	- -	-
**Above 60 minutes**	177	22	12.43	0.86	(0.55 to 1.33)	0.4941	1.24	(0.80 to 1.94)	0.3311
**Missing data**	359	39	10.86	0.75	(0.51 to 1.10)	0.1424	0.81	(0.56 to 1.17)	0.2701

N = number of patients/observations; Evts. =number of obese patients (BMI>=30);

% Evts. = percentage of overweight and obese patients; RR = risk ratio

(*) Associations analyzed using log-link binomial regression with robust variance estimates to adjust for within household clustering

Table S2. Multivariable association between Obesity and socioeconomic and behavioral characteristics in Maputo city, Mozambique (N=817)

				Univariable			Multivariable (**)		
**Covariate**	**N**	**Evts.**	**% Evts.**	**RR**	**95% CI**	**p-value**	**RR**	**95% CI**	**p-value**
**Gender**									
**Male***	346	11	3.18	1.00	- -	-	1.00	- -	-
**Female**	471	91	19.32	6.08	(3.36 to 11.00)	0.0000	5.67	(3.14 to 10.23)	<0.0001
**Age**									
**(per year**	-	-	-	1.05	(1.04 to 1.06)	0.0000	1.03	(1.02 to 1.05)	<0.0001
**Household wealth index score, quintiles**									

**Very low***	108	5	4.63	1.00	- -	-	1.00	- -	-
**Low**	146	21	14.38	3.11	(1.21 to 7.98)	0.0185	3.56	(1.44 to 8.82)	0.0061
**Medium**	174	24	13.79	2.98	(1.21 to 7.35)	0.0178	3.50	(1.46 to 8.36)	0.0049
**Medium/high**	192	17	8.85	1.91	(0.73 to 5.03)	0.1888	2.33	(0.93 to 5.86)	0.0709
**Highest**	197	35	17.77	3.84	(1.58 to 9.33)	0.0030	5.17	(2.19 to 12.20)	0.0002
**Education**									
**Primary or lower**	380	74	19.47	1.00	- -	-	1.00	- -	-
**Secondary or higher**	437	28	6.41	0.33	(0.22 to 0.50)	0.0000	0.60	(0.38 to 0.97)	0.0361
**Occupation**									
**Retired/unemployed***	160	24	15.00	1.00	- -	-	1.00	- -	-
**Housewife**	44	5	11.36	0.76	(0.30 to 1.89)	0.5514	0.58	(0.24 to 1.40)	0.2247
**Student**	132	4	3.03	0.20	(0.07 to 0.56)	0.0022	0.49	(0.18 to 1.37)	0.1748
**Employed**	403	59	14.64	0.98	(0.64 to 1.48)	0.9094	1.36	(0.93 to 1.97)	0.1106
**Missing data**	78	10	12.82	0.85	(0.43 to 1.71)	0.6561	1.04	(0.56 to 1.95)	0.8933

N = number of observations; Evts. = number of events; % Evts. = (N-events/N)*100; RR = risk ratio; 95% CI = 95% confidence interval

* reference stratum

** Uni- and multi-variable Poisson regression results; using robust variance estimates adjusted for clustering by household
